# Fatal Case of Enterovirus 71 Infection and Rituximab Therapy, France, 2012

**DOI:** 10.3201/eid1908.130202

**Published:** 2013-08

**Authors:** Somar Kassab, Tahar Saghi, Alexandre Boyer, Marie-Edith Lafon, Didier Gruson, Bruno Lina, Hervé Fleury, Isabelle Schuffenecker

**Affiliations:** Bordeaux University Hospital and Ségalen Bordeaux University, Bordeaux, France (S. Kassab, T. Saghi, A. Boyer, M.-E. Lafon, D. Gruson, H. Fleury);; French National Reference Center for Enteroviruses–Hospices Civils de Lyon, Bron, France (B. Lina, I. Schuffenecker)

**Keywords:** enterovirus 71, EV-71, rhomboencephalitis, rituximab, fatal case, France, viruses

**To the Editor:** Enterovirus 71 (EV-71) causes primarily asymptomatic or benign infections in children <5 years of age. However, it may cause severe and sometimes fatal neurologic complications, such as brainstem encephalitis and polio-like paralysis (*1*). Over the last 15 years, large outbreaks of EV-71 infection have been described in the Asia–Pacific region, associated with the regular emergence of new genetic lineages (*2*). Since the 1978 outbreak in Hungary, rare sporadic cases have been reported in Europe (*1*). In France, during 2000–2009, a total of 81 hospitalized patients with EV-71 infection were reported by the sentinel surveillance system, including 2 child deaths, 1 due to proven rhombencephalitis (*3*,*4*). 

We report here a fatal case of EV-71 rhombencephalitis in an immunocompromised adult who was receiving rituximab therapy. Rituximab is a chimeric anti-CD20 monoclonal antibody that is widely used for treating B-cell lymphoma and an increasing number of autoimmune diseases. Since rituximab became commercially available, several infectious side-effects for the drug have been reported, including hepatitis B reactivation, progressive multifocal leukoencephalopathy, and enteroviral meningoencephalitis (*5*). The first 2 cases of rituximab-associated enteroviral meningoencephalitis were reported in 2003 (*6*), and 5 additional cases have been reported to date (*7**,**8*).

In May 2012, a 66-year-old woman was hospitalized in the neurology unit of Bordeaux University Hospital with a 10-day history of fever, asthenia, and psychomotor retardation. She had no history of travel and had not been in close contact with sick persons. She had received a diagnosis of grade I follicular lymphoma 3 years earlier, and it had been treated with 6 cycles of R-CHOP (rituximab, cyclophosphamide, hydroxydaunorubicin, oncovin, prednisolone). Since July 2010, the lymphoma had been in remission, and she had been receiving maintenance therapy with rituximab since that time. The most recent rituximab infusion had been administered in March 2012. Her condition was treated initially with broad-spectrum antibiotics and acyclovir. Still, aphasia, facial paralysis, spastic movements, and consciousness disorders rapidly developed. On day 6, she was transferred to the intensive care unit for ventilatory support.

On patient’s admission, blood samples showed lymphopenia (0.64 × 10^3^ cells/mm^3^) and low immunoglobulin levels, i.e., IgG 4.5 g/L (reference range 6.75–12.8 g/L) and IgM 0.33 g/L (reference range 0.56–1.9 g/L). Three cerebrospinal fluid (CSF) samples were collected on days 1, 4, and 6. CSF leukocyte count rose from 5 to 89 cells/mm^3^, with lymphocytes from 24% to 95%, and protein levels rose from 0.68 to 1.03 g/L (reference range 0.15–0.45 g/L). CSF glucose level varied from 3.5 to 4.5 mmol/L (reference range 2.7–3.9 mmol/L). Enterovirus RNA was detected in the patient’s first 3 CSF samples and in CSF, stool specimens, and blood until 4 weeks after admission (Technical Appendix Table 1). PCR assays of the first 3 CSF samples were negative for JC polyomavirus, herpes simplex virus, varicella-zoster virus, cytomegalovirus, Epstein-Barr virus, human herpesvirus 6, adenovirus, and *Toxoplasma gondii*. Serologic tests for parvovirus B19, mumps virus, and measles virus were IgM negative. Samples were also negative for antibodies against Hu, Ri, Yo, and voltage-gated potassium channel antigens. All bacterial cultures were negative. No evidence for central nervous system infiltration by lymphoma cells was found, on the basis of CSF cytology.

Results of brain magnetic resonance imaging (MRI) scans performed on days 2 and 6 were normal, despite the patient’s consciousness disorders (Figure, panel A). However, on day 13, MRI scans showed bilateral and symmetric T2 and FLAIR hypersignals in the medulla, the pons, and the mesencephalon, compatible with rhomboencephalitis (Figure, panel B). On day 24, the MRI scan showed a supratentorial extension involving white matter, the insular cortex, and basal ganglia (Figure, panel C). The patient’s neurologic condition deteriorated progressively, and she died of enteroviral rhomboencephalitis 32 days after admission. 

The EV associated with the rhomboencephalitis was identified as an EV-71 genogroup C2 isolate by 1D gene complete sequencing and phylogenetic analysis (Technical Appendix Figure; Technical Appendix Table 2). The 1D gene sequences determined from cerebrospinal fluid and fecal specimens from the patient showed 95%–97% nucleotide homology and clustered with 1D gene sequences from strains detected during 2006–2012 in France, the Netherlands, Germany, Spain, Canada, United Kingdom, and Singapore.

Only 7 cases of rituximab-associated EV encephalitis have been reported in the literature. Of the case-patients, 3 died from enteroviral meningoencephalitis, 1 showed partial neurologic improvement but died later from another infection (not specified), 2 suffered permanent sequelae, and 1 recovered completely (*6*–*8*).

The first case of fatal rituximab-associated EV-71 encephalitis was reported in Australia in 2011 (*8*). The Australian patient and the French patient reported here were adults, although most EV-71 encephalitis cases have been described in children (*1*). Neither adult patient exhibited neurogenic pulmonary edema. Both cases were associated with genogroup C2 EV-71 strains that were closely related to those that have been detected in recent years in Europe and worldwide (*3*).

Because invasive EV infections have been described in adults with hereditary or congenital defects in B-lymphocyte function, humoral immunity is likely to play a key role in EV infection control (*9*). Passive protection against lethal EV-71 infection in newborn mice by neutralizing antibodies is another convincing argument that the antibody-mediated response is critical (*10*). Thus, because rituximab is associated with long-lasting B-cell depletion and, in some patients, a decrease in immunoglobulin, it may lead to an increased risk for EV encephalitis. Although EV encephalitis seems to be rare in patients who receive rituximab treatment, cases may have been underdiagnosed. To detect this condition and prevent possible deaths, physicians should routinely screen for EV RNA in patients receiving anti-CD20 therapy who have neurologic symptoms and should consider the early administration of immunoglobulin.

**Figure:**
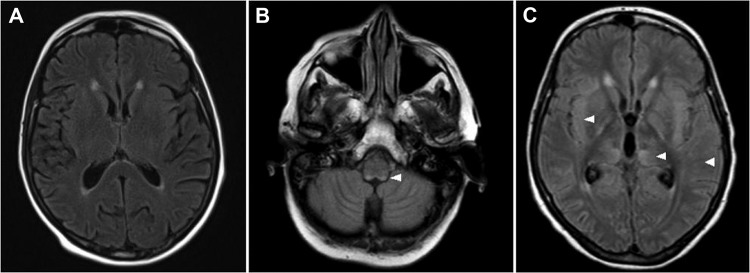
Magnetic resonance imaging axial flair sequence of brain of 66-year-old woman with fatal encephalitis, Bordeaux, France, 2012. A) No hypersignal at day 6. B) Bilateral posterior hypersignals in the medulla at day 13. C) Bilateral supratentorial hypersignals at day 24 in the cortex, the white matter, and the basal ganglia. Hypersignals are indicated by white arrowheads.

Technical Appendix Enterovirus RNA detected in the patient’s cerebrospinal fluid, blood, and stool specimens and identified as an EV-71 genogroup C2 isolate and phylogenetic analysis of complete viral protein 1 coding sequences of enterovirus 71 strains.
