# Supplementary material for: Fatal Case of Enterovirus 71 Infection and Rituximab Therapy, France, 2012
Source: Emerg Infect Dis. 2013 Aug;19(8):1345–7. doi: 10.3201/eid1908.130202 (PMC3739532; doi:10.3201/eid1908.130202)
Supplement: Technical Appendix — Enterovirus RNA detected in the patient’s cerebrospinal fluid, blood, and stool specimens and identified as an EV-71 genogroup C2 isolate and phylogenetic analysis of complete viral protein 1 coding sequences of enterovirus 71 strains. [file 13-0202-Techapp-s1.pdf]

Article DOI: http://dx.doi.org/10.3201/eid1908.130202

Fatal Case of Enterovirus 71 Infection and Rituximab Therapy, France, 2012

Technical Appendix

Technical Appendix Table 1. PCR results showing enterovirus in CSF, blood, and fecal specimens*

Date specimen obtained, 2012	CSF	Blood	Stool
May 26	+ (35)		
May 29	+ (32)		
May 31	+ (37)	+ (32)	
Jun 4			+ (33)
Jun 6	+ (35)		
Jun 12	+ (24)		+ (29)
Jun 25	+ (28)		

*The cycle threshold is indicated in parentheses. CSF, cerebrospinal fluid.

Technical Appendix Table 2. Enterovirus 71 strains selected for phylogenetic analysis

Genogroup	Origin	Year	Strain	GenBank no.	Diagnosis
A	USA	1970	BrCr	U22521	Meningitis
B1	Hungary	1978	Hungary-78	AB059815	NA
B2	USA	1988	8149-AL-88 55E	AF135907	NA
B3	Malaysia	1997	0884-MAA-97	AY207645	NA
B4	Australia	2000	9918-SYD-00	AY940106	NA
B5	Sarawak	2003	SB12869-SAR-03	AY905545	NA
C1	USA	1987	0915-MA-87	AF009549	NA
C3	Korea	2001	KOR-EV71-01	AY125966	NA
C4	China	2000	F1-CHN-00	AB115490	NA
C5	Vietnam	2005	999T/VNM/05	AM490163	NA
C2	Australia	1999	6F/AUS/6/99	DQ381846	Meningitis
C2	Canada	2007	EV053_07	HQ647175	NA
C2	France	2000	GRE1244_FRA00	FN598728	NA
C2	France	2006	LYO03126_FRA06	FN598754	Meningitis
C2	France	2007	BRE324_FRA07	FN598762	Fatal rhomboencephalitis with neurogenic pulmonary edema
C2	France	2008	NAN257_FRA08	FN598776	NA
C2	France	2009	ANG261_FRA09	FN598777	Myelitis, diarrhea
C2	France	2010	LYO29244_FRA10	This study	Neonatal fever
C2	France	2011	ROU09_FRA11	This study	Neonatal fever, rash
C2	France	2012	LYO40162_FRA12	This study	Acute cerebellar ataxia
C2	France	2012	CSF-BOR1717_FRA12	This study	Fatal rhomboencephalitis
C2	France	2012	FECES-BOR1718_FRA12	This study	Fatal rhomboencephalitis
C2	Great Britain	1999	EP/7414/1999	AM939585	NA
C2	Great Britain	2006	STH/MCN/2006	AM939597	Fatal panencephalitis
C2	Germany	2008	STU543078_GER08	FN649263	NA
C2	Netherlands	2000	365	AB491218	NA
C2	Netherlands	2007	3692	AB575942	NA
C2	Netherlands	2008	265	AB491220	NA
C2	Netherlands	2010	10118	AB575948	NA
C2	Singapore	2008	NUH0075/SIN/08	FJ172159	NA
C2	Spain	2008	ESP08/54678	FR798002	NA
C2	Taiwan	1998	1245a/98/tw	AF176044	HFMD
C2	USA	1997	2355-OK-97	AF135942	NA

*ARDS, acute respiratory distress syndrome; NA, not available; HFMD, hand, foot and mouth disease.

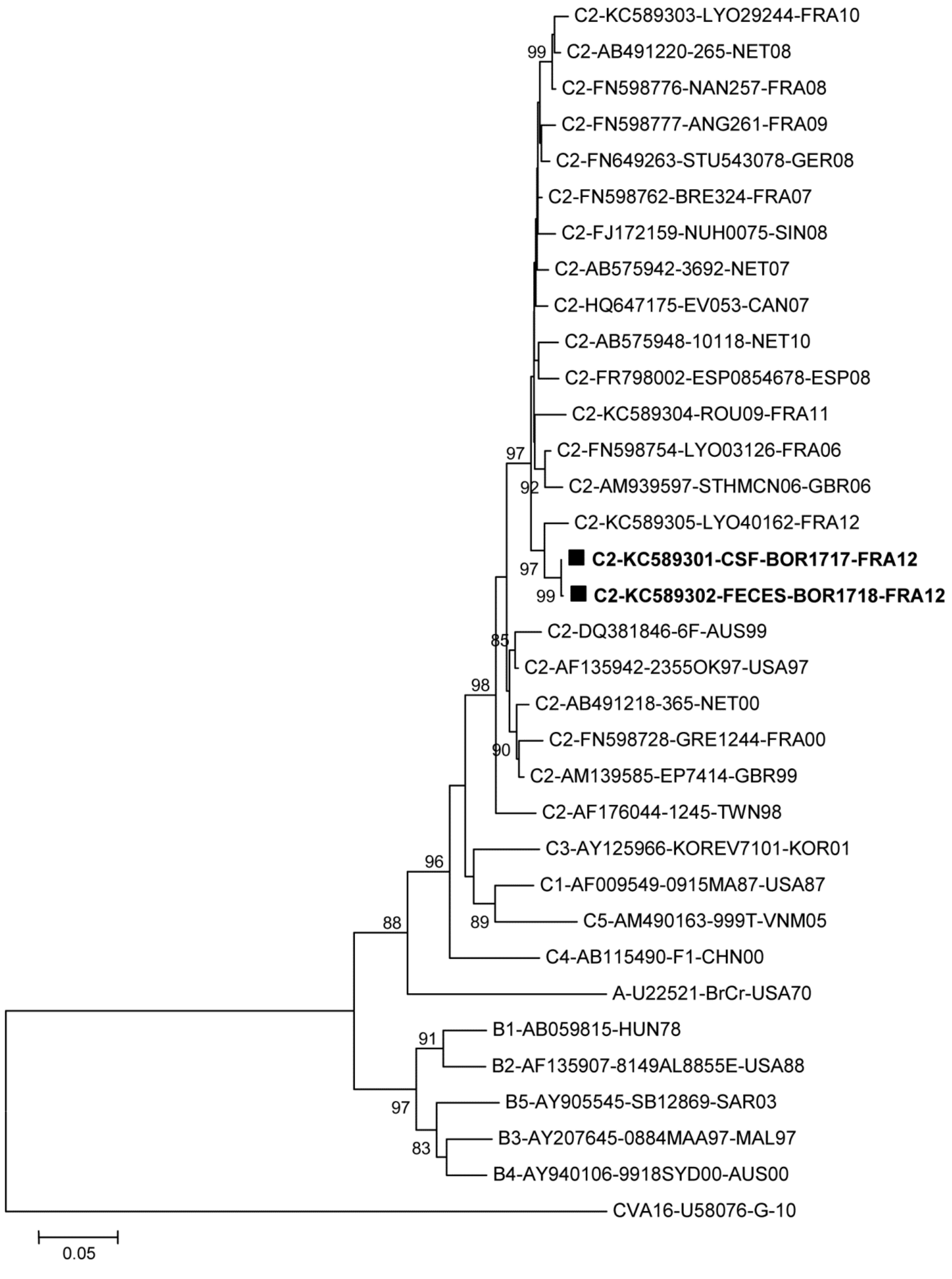

Technical Appendix Figure. Phylogenetic analysis of complete viral protein 1 coding sequences of enterovirus (EV) 71 strains. The analysis included the sequences determined from the cerebrospinal and stool specimens collected from the patient with fatal rhombencephalitis (shown by a square), 8 selected genogroup C2 sequences from French strains (detected between 2000 and 2012), and 23 Gen-Bank-

selected worldwide strains. The prototype coxsackievirus A16 strain was used as an outgroup virus. Genetic distances were calculated with the Maximum Composite Likelihood model of evolution. The tree was constructed by the neighbor-joining method, using MEGA5, and validated using 1,000 bootstrap pseudo-replicates. Designation of strains was as follows: subgenogroup of the strain/GenBank accession number/lab number of the isolate/3-letter country code/ year of detection.
